# Therapeutic roles of telocytes in OVA‐induced acute asthma in mice

**DOI:** 10.1111/jcmm.13199

**Published:** 2017-05-19

**Authors:** Ling Ye, Dongli Song, Meiling Jin, Xiangdong Wang

**Affiliations:** ^1^ Department of Respiratory Medicine Zhongshan Hospital Fudan University Shanghai China; ^2^ Zhongshan Hospital Institute of Clinical Science Fudan University Shanghai China

**Keywords:** asthma, telocytes, mesenchymal stem cells, airway inflammation, airway hyper‐responsiveness

## Abstract

Telocytes (TCs) newly discovered as the mesenchyme‐derived interstitial cells were found to have supportive effects on mesenchymal stem cells (MSCs). The present study aimed at investigating effects of TCs or TCs gathered with MSCs on experimental airway inflammation and hyper‐responsiveness. The TCs were isolated from the lung tissue of the female BALB/c mice. The ovalbumin (OVA)‐induced asthma model was established. TCs (1 × 10^6^/2 × 10^6^) and/or MSCs (1 × 10^6^) were injected through mice tail vein for consecutive three days before OVA excited the mice. This study at first demonstrated that the transplantation of TCs could improve allergen‐induced asthma by obviously inhibiting airway inflammation and airway hyper‐responsiveness preclinically, with the down‐regulation of Th2‐related cytokine IL‐4, transcription factor GATA‐3 and Th2 cell differentiation, while up‐regulation of Th1‐related cytokine IFN‐γ, transcription factor T‐bet and Th1 cells proliferation in asthma, just like MSCs. Co‐transplantation of TCs with MSCs showed better therapeutic effects on experimental asthma, even though the therapeutic effects of TCs alone were similar to those of MSCs alone. TCs and the combination of TCs with MSCs could improve the airway inflammation and airway hyper‐responsiveness and can be a new alternative for asthma therapy.

## Introduction

Telocytes (TCs) are newly discovered as the mesenchyme‐derived interstitial cells in recently years 1, of which the typical long prolongations named as the telopode with the vital morphological feature to connect to other cells 2. TCs can form a number of three‐dimensional networks with other cells through multipoints and local close contacts of telopode 3. Biological functions of TCs can be performed in patterns of juxtacrine and paracrine by producing extracellular vesicles to transfer the signalling materials to neighbouring cells 4, 5. TCs play an important role in maintenance and regulation of the microenvironmental homoeostasis through the intercellular signalling transduction, neurotransmitter transmission, immune regulation or immune surveillance 6. Moreover, TCs comprise Oct4‐positive cell, which express pluripotency‐related genes 7.

Pathophysiological changes of TCs quantity or function were noticed in a large number of diseases, such as myocardial infarction, heart failure, inflammatory bowel disease, liver fibrosis and systemic sclerosis 8, 9, 10, 11. Mesenchymal stem cells (MSCs) were found to experimentally alleviate the airway inflammation and decrease airway hyper‐responsiveness 12 and secrete soluble cytokines through paracrine to regulate T cells to treat asthma 13, 14. Depending on the role of TCs on the immune regulation, we speculate that TCs may play a role in asthma like MSCs. TCs are widely distributed in the lung tissue, but the study on the effect of TCs in asthma is rare. This study aimed to investigate whether TCs could be used to treat asthma in animal models.

## Materials and methods

### Animals

Female BALB/c mice, weighing 18–22 g (6–8 weeks), were provided by Animal Facility in Biomedical Research Center of Zhongshan Hospital, Fudan University. This study was approved by the Fudan University Ethical Committee for animal experiments.

### Lung TCs isolation and identification

The isolation and identification of lung TCs were described by previous study 15. Briefly, mice were anaesthetized, of whom the lung tissues were isolated and digested by digestive fluid. After neutralizing digestive fluid, the fluid was filtered by 40 μm cell strainer and then centrifuged at 400 g for 5 min. Cells were collected and cultured in culture bottles in incubator (37°C, 5% CO_2_). The supernatant was moved into new culture bottles 30 min. after cell adhesion on the plate. Cells were selected, purified and further amplified to meet the need for the experiment. The TCs were identified according to the morphology and immunofluorescent staining using mouse anti‐cKit antibody, rat anti‐vimentin antibody and rabbit anti‐CD34 antibody (1:200 dilution; Abcam, Cambridge, UK). After incubated overnight at 4°C with the first antibodies diluted in 1% bovine serum albumin (BSA) in PBS, the slides were washing in PBS for three times. Then, sections were incubated with PE conjugated antimouse secondary antibodies, PE conjugated anti‐rat secondary antibodies or FITC conjugated anti‐rabbit secondary antibodies according to the manufacturer (1:150 dilution; Abcam). DAPI was used to mark nuclear according to the manufacture (Abcam).

### MSCs isolation and identification

The femur and tibia of mice were isolated under sterile condition. The osteoepiphysis of femur and tibia were cut off, and the marrow cavities were washed with DMEM/F12 medium. The lavage fluid was centrifuged at 100 g for 10 min., and cells were collected and cultured in incubator (37°C, 5% CO_2_), after red blood cells were lysed. The medium was changed every 48 hrs, and MSCs were identified according to the morphology and flow cytometry for cell markers such as CD90‐PE, CD105‐PE and CD34‐PE (1:50 dilution; BD Biosciences, Palo Alto, CA, USA).

### Induction of asthma models

The mice were intraperitoneally injected with 100 μl 0.02% ovalbumin (OVA) and 100 μl Al(OH)_3_ suspension (Sigma‐Aldrich, St. Louis, MO, USA) at 1, 8 and 15 days (d). At 25–28 days, the mice were per‐nasally instilled with 50 μl 0.4% OVA. The airway reactivity was measured, and specimens were collected 24 hrs after the last provocation. At 22–24 days, the mice were intravenously injected with the suspension of TCs alone, MSC alone, or the combination for 3 consecutive days.

BALB/c mice were randomly divided into seven groups (*n* = 10/group): (1) animals were intraperitoneally sensitized with OVA, intratracheally provoked with vehicle and intravenously treated with vehicle as negative controls (PBS); (2) animals sensitized with OVA, provoked with vehicle and treated with TCs at 10^6^ per day (PBS + TCs); (3) animals sensitized and provoked with OVA, and treated with vehicle as positive controls (OVA); (4) animals sensitized and provoked with OVA, and treated with TCs at 10^6^ per day (L‐TCs); (5) animals sensitized and provoked with OVA, and treated with TC at 2 × 10^6^ per day (H‐TCs); (6) animals sensitized and provoked with OVA, and treated with MSCs at 10^6^ per day (MSC); and (7) animals sensitized and provoked with OVA, and treated with the combination of TCs and MSCs at 10^6^ per day, respectively (TCs + MSC) (Fig. S1).

### Detection of migration to the lungs of TCs and MSC

An additional experiment was designed to confirm the migration of TCs and MSCs into the lung after the intravenous injection of the living TCs labelled with PKH26 (Red) and MSC with 5(6)‐(N‐succinimidyloxycarbonyl)‐3′,6′,O,O’‐diacetylfluorescein (CFSE) (Green) (Sigma‐Aldrich) (*n* = 4 animals/group). Frozen sections of lungs were prepared to observe the distribution of TCs and MSCs using Leica TCS SP5 confocal microscope (Leica Microsystems, Wetzlar, Germany).

### Measurement of bronchial hyper‐responsiveness

The airway hyper‐responsiveness 24 hrs after the last provocation of OVA was measured using the FinePointe Resistance and Compliance (Buxco, Wilmington, NC, USA) as lung resistance (RL). The concentrations of methacholine for provocation were 6.25, 12.5 and 25 mg/ml, respectively. The percentage of RL value and basal RL value were used to reflect the airway responsiveness after the provocation of methacholine.

### Pathological evaluations

Pathological changes of lung injury were evaluated according to Underwood's standard of lung histopathological scoring 16. The hyperplasia and hypertrophy of the goblet cells in the airway were assessed with PAS. The processes were separately conducted by two pathologists, and the average values were used for the results.

### Assay of airway inflammation

Leucocytes in the bronchoalveolar lavage fluid (BALF) were harvested, stained with Wright‐Giemsa dye and counted after centrifugation at 200 g for 15 min. (4°C). Levels of inflammatory mediators, for example interleukin (IL)‐4, interferon (IFN)‐γ, transforming growth factor‐beta (TGF‐β) (Sigma‐Aldrich) and OVA‐specific IgE (Bio‐Rad, Hercules, CA, USA) were measured with ELISA kits as suggested by the manufactory.

### Isolation and validation of spleen cells

An additional experiment was designed and performed to evaluate CD4+ T‐cell phenotypes. The mouse spleen was acquired under sterile condition, cut into small pieces and filtered with 70‐μm strainer to remove the capsule and connective tissue. The cell supernatant was collected and centrifuged at 1500 rpm for 10 min., with thrice washes atTris‐NH4 cl. FACSAria II flow cytometry (BD Biosciences, San Diego, CA, USA) was used to test spleen CD4+ T‐cell subgroups in animals mentioned above. The CD4+ T cells were isolated by flow cytometry and then were labelled with IFN‐γ‐PE, IL‐4‐PE and Foxp3‐PE antibodies (BD). The proportion of CD4 + IFN‐γ+ T cells, CD4 + IL‐4+ T cells and CD4 + Foxp3+ T cells was accounted. The mRNA expression of T‐bet, GATA‐3 and Foxp3 in lung tissues harvested from various groups was measured on basis of gene probes as listed in Table 1, using Rotor‐Gene 3000 fluorescence ration PCR instrument (Corbett Research, Sydney, Australia).

**Table 1:** Primer sequences for the RT‐PCR

Gene	Forward primer	Reverse primer
GAPDH	5′‐ AATGGGAGAACAGGGGAAAT‐3′	5′‐ ACCGAAGAACAACGAGGAGA‐3′
T‐bet	5′‐GTTCAACCAGCACCAGACAGAG‐3′	5′‐TGGTCCACCAAGACCACATC‐3′
GATA‐3	5′‐GGATGTAAGTCGAGGCCCAAG‐3′	5′‐ATTGCAAAGGTAGTGCCCGGTA‐3′
Foxp3	5′‐AGGAGAAAGCGGATACCA‐3′	5′‐TGTGAGGACTACCGAGCC‐3′

### Statistics

Data were analysed using IBM SPSS Statistics 20 (Chicago, IL, USA). Each point corresponds to the mean ± S.E.M. Statistical differences between two groups were determined by *t*‐test, and statistical differences among more than two groups were determined using anova. *P* < 0.05 was considered significant.

## Results

### The identification and distribution of TCs

**Figure 1:**
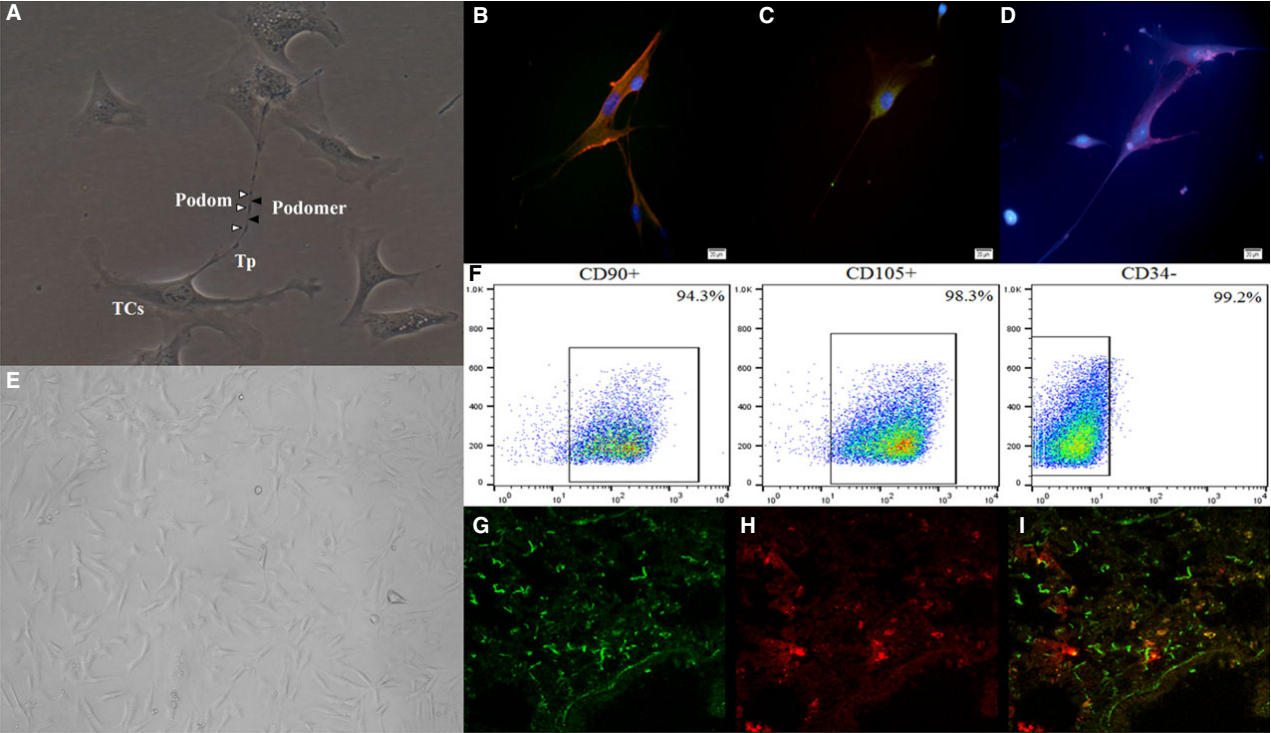
The identification and lung distribution of telocytes (TCs) and mesenchymal stem cells (MSCs). (**A**) The morphology of TCs under the light microscope. The bead‐like extending cellular process was named as telopode (Tp), with alternation of podoms (▵) and podomers (▲) (×200). (**B**) vimentin (Red) and CD34 (Green) fluorescence staining was positive. (**C**) CD34 (Green) fluorescence staining was positive. (**D**) c‐kit (Red) fluorescence staining was positive (×200). (**E**) The morphology of MSCs under the light microscope (×200). (**F**) The surface antigen CD90 and CD105 of MSCs were positive, and CD34 was negative. The mice lung tissue of TCs + MSC group, (**G**) CFSE‐labelled MSCs (Green); (**H**) PKH26‐labelled TCs (Red); (**I**) The merged image of two cells (*n* = 4/group).

The lung‐origin TCs were identified with the morphological features (Fig. 1A) combined with relatively special cellular surface biomarkers CD34, c‐kit and vimentin (Fig. 1B–D), especially the extending cellular process—telopode which did not appear in MSCs (Fig. 1E). The flow cytometry demonstrated that the surface biomarkers CD90 and CD105 were positive and CD34 was negative in MSCs (Fig. 1F). Lung distribution of PKH26‐labelled TCs and/or CFSE‐labelled MSC was traced after the intravenous injection and shown in Figure 1G–I. The most of labelled TCs were located along the alveolar wall within the capillaries and some in the interstitial space where some TCs gathered with MSCs.

### Roles of TCs in the airway hyper‐responsiveness

**Figure 2:**
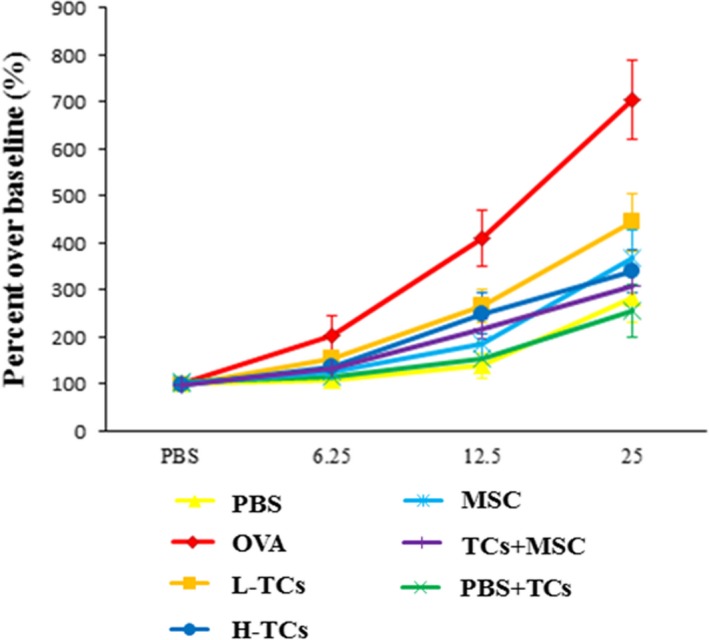
The effect of telocytes (TCs) on the airway hyper‐responsiveness. When stimulated with the concentration of methacholine was 6.25 mg/ml, the airway hyper‐responsiveness was similar between L‐TCs group and ovalbumin (OVA) group; the airway hyper‐responsiveness of the H‐TCs group, mesenchymal stem cells (MSC) group and TCs + MSC group was lower than OVA group. When stimulated with the concentration of methacholine was 12.5 and 25 mg/ml, the airway hyper‐responsiveness of four groups was lower than OVA group (*n* = 4/group).

The similar levels of airway hyper‐responsiveness were found in OVA animals treated with vehicle and L‐TCs after the exposure to the low concentration of methacholine at 6.25 mg/ml (*P* = 0.14, Fig. 2). Treatments with H‐TCs or MSCs alone or the combination (TCs + MSC) significantly reduced airway hyper‐responsiveness to OVA, as compared with those with vehicle (*P* < 0.05 or less, respectively). When stimulated with the concentration of methacholine is 12.5 mg/ml, the airway hyper‐responsiveness of OVA animals treated with L‐TCs, H‐TCs, MSCs or TCs + MSC was significantly lower than those treated with vehicle (*P* < 0.05 or less, respectively) after the exposure of middle concentration of methacholine at 12.5 or 25 mg/ml, respectively. At exposure to high concentration of 25 mg/ml, the level of airway hyper‐responsiveness in OVA animals treated with TCs + MSCs was lower than those treated with L‐TCs alone (*P* = 0.004).

### Roles of TCs in airway inflammation and mucus secretion

**Figure 3:**
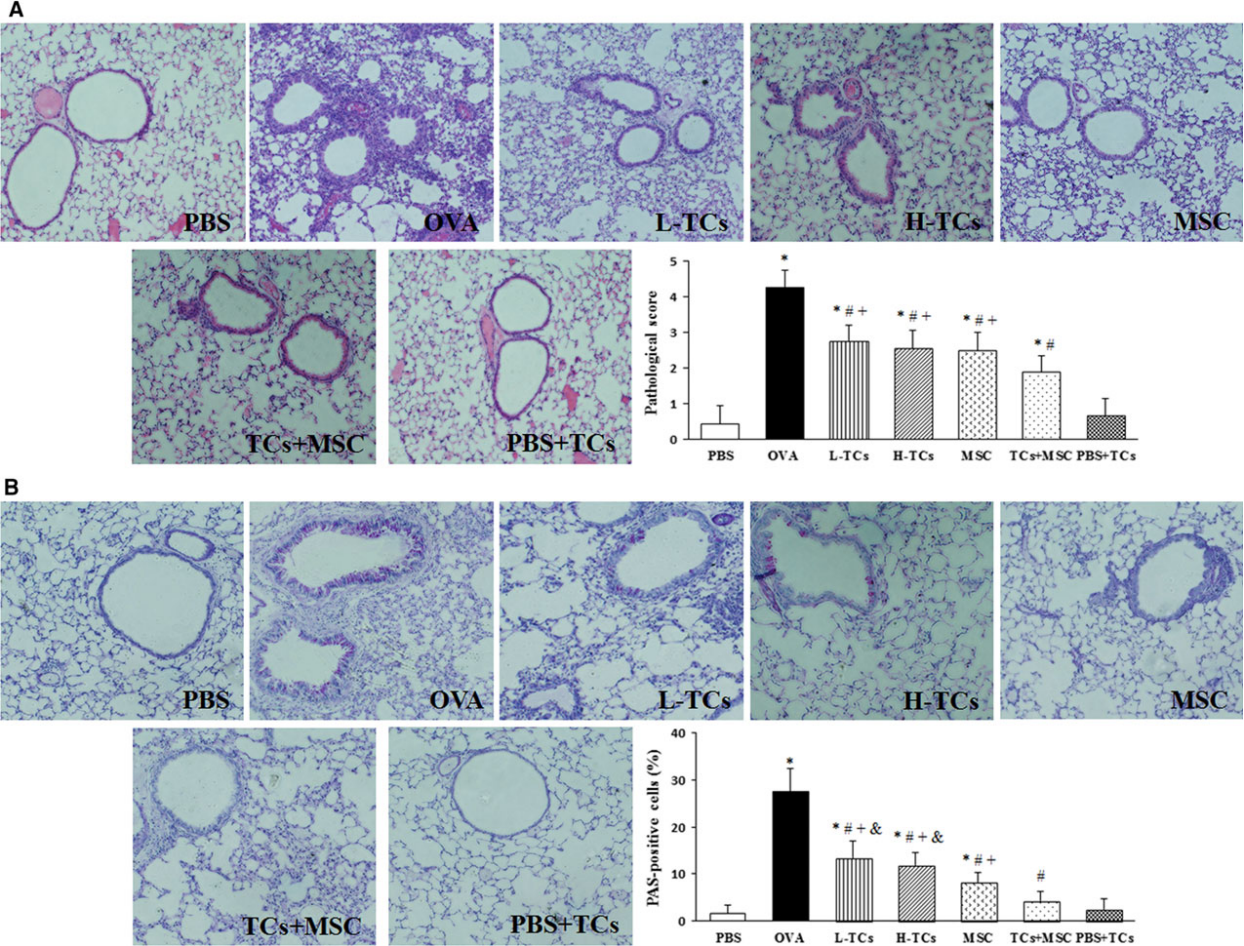
The effect of telocytes (TCs) on the airway inflammation and mucus secretion. TCs significantly decreased the airway inflammation (**A**) and mucus secretion (**B**). There was no statistically difference between L‐TCs group and H‐TCs group. TCs+ mesenchymal stem cells (MSC) group had a stronger inhibitory effect on the airway inflammation and mucus secretion (*n* = 6/group) (×200). *P < 0.05 *versus* PBS group; #*P* < 0.05 *versus* ovalbumin (OVA) group; +*P* < 0.05 *versus* TCs + MSC group; &*P* < 0.05 *versus* MSC group.

**Figure 4:**
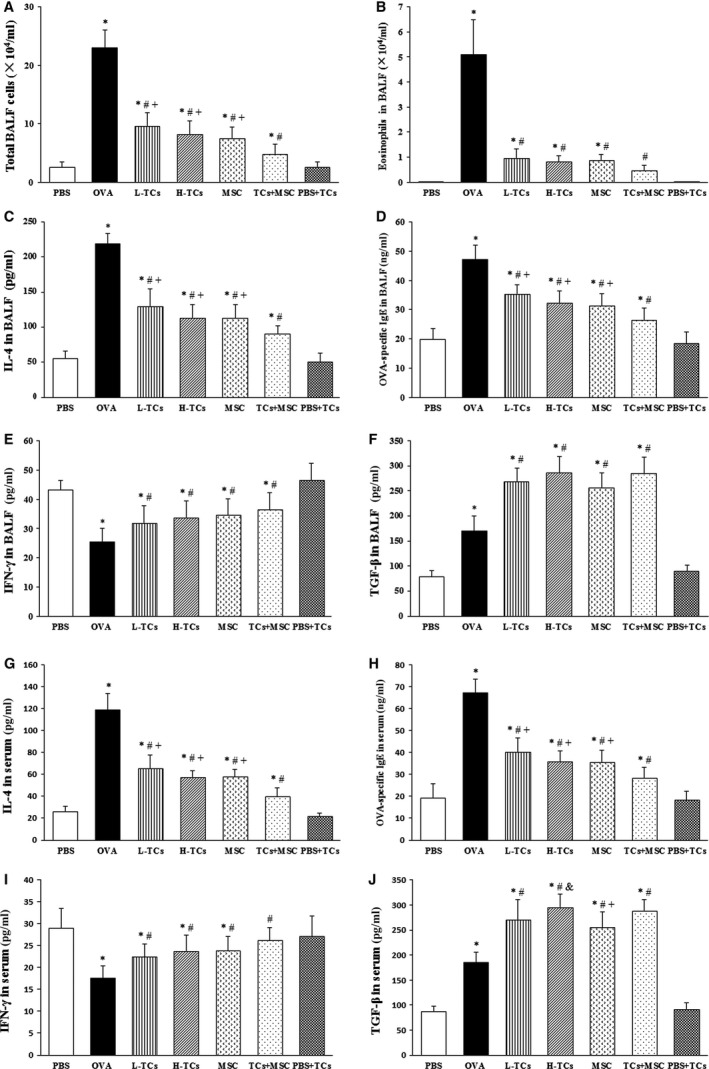
The effect of telocytes (TCs) on the cell count in bronchoalveolar lavage fluid (BALF) and inflammatory cytokinesis BALF and serum. TCs significantly decreased the total cell count (**A**) and eosinophils count (**B**) in BALF, just like mesenchymal stem cells (MSC). TCs obviously decreased the IL‐4 (**C**,** G**) and ovalbumin (OVA)‐specific IgE (**D**,** H**) while increased the IFN‐γ (**E**,** I**), TGF‐β (**F**,** J**) in BALF and serum. Combination TCs with MSC could more obviously impaired Th2‐related inflammatory response (*n* = 6/group). *P < 0.05 *versus* PBS group; #*P* < 0.05 *versus* OVA group; +*P* < 0.05 *versus* TCs + MSC group; &*P* < 0.05 *versus* MSC group.

The score of pathological changes in OVA animals treated with L‐TCs, H‐TCs, MSC or TCs + MSC was significantly lower than those treated with vehicle, while still higher than animals provoked and treated with vehicle (*P* < 0.05 or less, respectively, Fig. 3A). The score of OVA animals treated with TCs + MSC was lower than those treated with L‐TCs, H‐TCs or MSCs alone (*P* < 0.05 or less, respectively). Figure 4B demonstrated the proportion of airway goblet cells to epithelial cells in OVA animals treated with vehicle was significantly higher than in those treated with L‐TCs, H‐TCs, MSCs or TCs + MSC (*P* < 0.05 or less, respectively). The percentage of airway goblet cells in OVA animals treated with TCs + MSC was significantly lower than in those treated with either TCs or MSCs alone, respectively (*P* < 0.05 or less, respectively), similar to the levels of animals sensitized with OVA and provoked with vehicle (*P* = 0.149). The percentage of airway goblet cells in OVA animals with MSC group was significantly lower than in those treated with TCs at low and high concentrations (*P* = 0.004, *P* = 0.046, respectively).

### Roles of TCs in OVA‐induced inflammation

The total leucocytes (Fig. 4A) and eosinophils (Fig. 4B) in BALF significantly increased in OVA animals treated with vehicle, as compared with those provoked and treated with vehicle (*P* < 0.05), which was prevented by treatments with L‐TCs, H‐TCs, MSCs or TCs + MSC (*P* < 0.05 or less, respectively), although the levels are still higher than in those provoked and treated with vehicle. The number of total leucocytes in BALF of OVA animals treated with TCs + MSC was significantly lower than those treated with either TCs or MSCs alone (*P* < 0.05 or less, respectively). The number of eosinophils in animals with TCs + MSC was not different from those provoked and treated with vehicle (*P* = 0.155), while the number of total leucocytes was still higher (*P* = 0.049).

Ovalbumin provocation significantly increased levels of IL‐4 and OVA‐specific IgE and decreased IFN‐γ proteins in BALF (Fig. 4C–E, respectively) and serum (Fig. 4G–I, respectively), which were prevented by treatment with TCs, MSCs or TCs + MSC. Levels of TGF‐β in BALF (Fig. 4F) and serum (Fig. 4J) significantly increased after the treatment with TCs, MSCs or TCs + MSC, as compared with OVA animals treated with vehicle (*P* < 0.05 or less, respectively).

### The effect of TCs on the proportion of spleen CD4+ T‐cell subgroup

**Figure 5:**
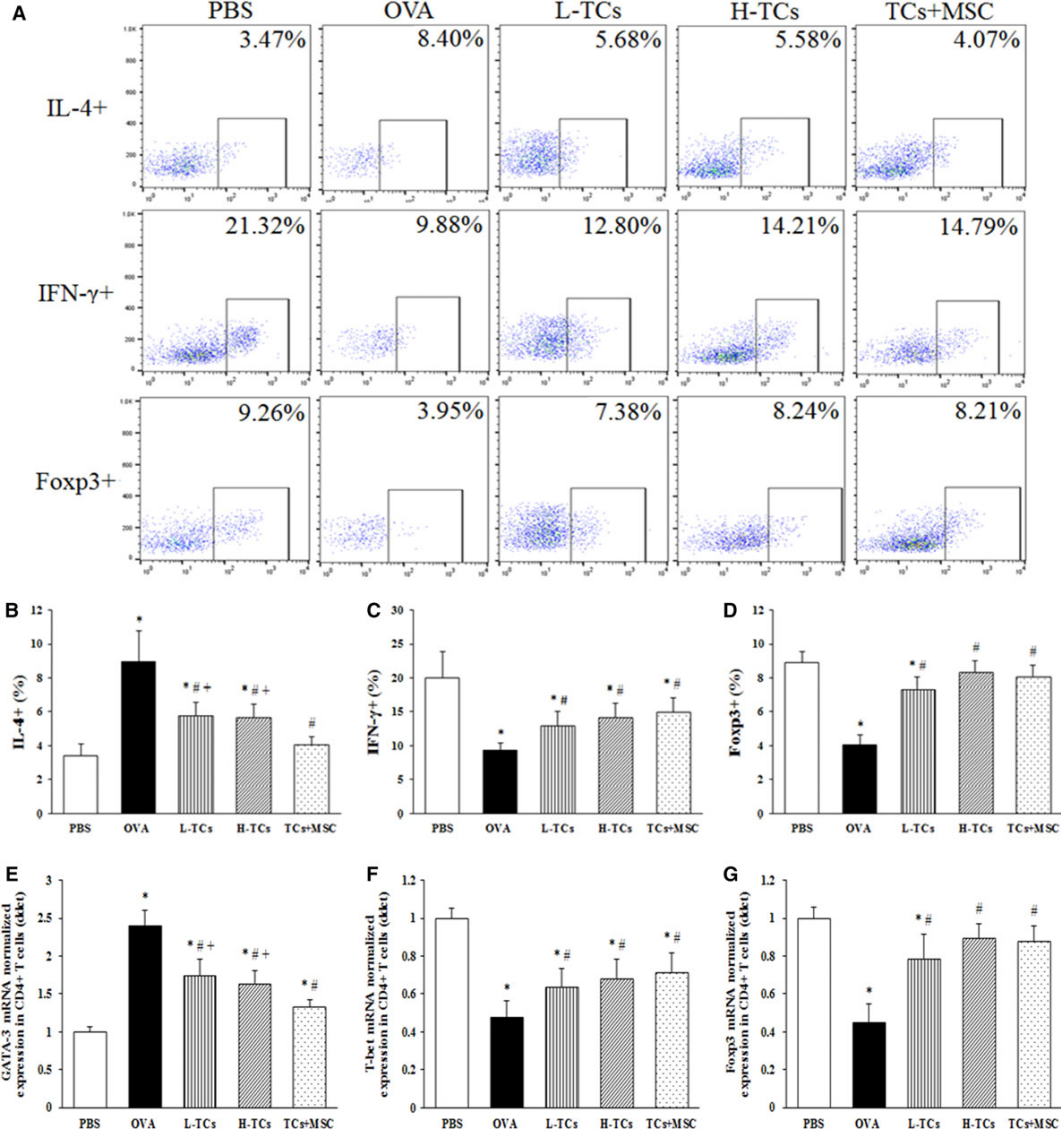
The effect of telocytes (TCs) on spleen CD4+ T cell. Flow cytometry showed that the proportion of spleen CD4 + T‐cell subgroup (**A**). TCs significantly decreased the proportion of CD4 + IL‐4+ T cells (**B**) and increased the proportion of CD4 + IFN‐γ+ T cells (**C**) and CD4 + Foxp3+ T cells (**D**). TCs could significantly decrease GATA‐3 mRNA (**E**) and increase T‐bet mRNA (**F**) and Foxp3 mRNA (**G**) in spleen CD4+ T cells. There was no statistical difference between TCs group in two doses. Combination TCs with mesenchymal stem cells (MSC) could more obviously decreased the proportion of CD4 + IL‐4+ T cells and the GATA‐3 expression (*n* = 6/group). *P < 0.05 *versus* PBS group; #*P* < 0.05 *versus* ovalbumin (OVA) group; +*P* < 0.05 *versus* TCs + MSC group.

The proportion of spleen CD4 + IL4+ T cells was significantly increased in OVA animals treated with vehicle, while CD4 + IFN‐γ+ or CD4 + Foxp3+ T cells decreased, as shown in Figure 5A–D, which were improved by the treatment with L‐TCs, H‐TCs or TCs + MSC (*P* < 0.05 or less, respectively). The proportion of spleen CD4 + IL4+ T cells in OVA animals treated with TCs + MSCs was significantly lower, as compared with those with L‐TCs or H‐TCs alone (*P* = 0.036, *P* = 0.048, respectively).

mRNA expression of GATA‐3 (Fig. 5E) up‐regulated, while T‐bet (Fig. 5F), and Foxp3 (Fig. 5G) down‐regulated on spleen CD4+ T cells of OVA animals treated with vehicle, which were improved with treatment with L‐TCs, H‐TCs or TCs + MSC. GATA‐3 mRNA in OVA animals treated with TCs + MSC was obviously lower than those with L‐TCs or H‐TCs alone (*P* = 0.011, *P* = 0.042, respectively).

## Discussion

Preclinical studies demonstrated that MSC could alleviate airway inflammation and airway hyper‐responsiveness in allergic models 12. The present study provided the initial evidence that the intravenous transplantation of TCs could improve lung inflammation and hyper‐responsiveness in experimental asthma and the combination of TCs with MSCs showed better therapeutic effects on allergen‐induced lung inflammation. We also found the therapeutic effects of TCs were similar to MSCs on airway hyper‐responsiveness, infiltration of inflammatory cells, production of inflammatory mediators, and goblet cell hypertrophy and hyperplasia.

Th1/Th2 imbalance was considered as the pathogenesis of asthma, and the decreased quality and quantity of Treg cells were suggested to play the critical roles in the development of asthma 17, 18. MSCs were found to up‐regulate Treg cells and inhibit immune hyper‐responses through secreted TGF‐β 19, 20, and influence and reverse the Th1/Th2 imbalance by promoting Th1 cells differentiation through secreted IFN‐γ 21. Results from the present study demonstrated TCs could influence the balance of Th1/Th2 by the down‐regulation of Th2‐related cytokine IL‐4, transcription factor GATA‐3 and Th2 cell differentiation, and up‐regulation of Th1‐related cytokine IFN‐γ, Th1‐related transcription factor T‐bet and Th1 cells proliferation. With therapeutic effects of TCs on experimental asthma, we found that TCs could increase the systemic and local production of TGF‐β and altered the proportion of Treg cells and increase the transcription factor Foxp3 mRNA. It suggested that the interaction between TCs and Treg cells can be a critical part of cellular mechanisms by which TCs improved allergen‐induced airway inflammation and hyper‐responsiveness.

Inflammatory mediators play regulatory effects on the differentiation of CD4+ T‐cell subgroups, for example the promotion of IFN‐γ in the differentiation of naive CD4+ T cells into Th1 cells. Moreover, TGF‐β plays a key role in Treg differentiation. Our results showed that TCs could increase the TGF‐β and IFN‐γ in asthmatic animals. TCs were found to release extracellular vesicles with mediators to transfer signalling to neighbouring cells by paracrine 22. It is possible that TCs improved allergen‐induced airway inflammation and hyper‐responsiveness by producing TGF‐β to promote the differentiation of naive CD4+ T cells into Treg cells, or secreting IFN‐γ to promote the differentiation of naive CD4+ T cells into Th1 cells.

The previous studies found that there were many TCs distributed around the stem cell niches in organ tissue 23, 24. Such niches are full of adult stem cells in various organ tissues and play an important role in stem cells survival and differentiation 25, 26. It may be easier to understand that TCs can surround the stem cells through telopode and form the juxtacrine and paracrine to supply nutrition support or signalling transduction on stem cells, as called ‘stem cell helper cells’ 27. In addition, partial genes of TCs were found to play roles in cell signalling by analysing the genetic profile of murine lung TCs 28, 29. However, our findings indicate that transplanted TCs may interact with MSCs mainly through the altered microenvironments from TCs or MSCs and the intercellular communication of mediators, as the majority of transplanted TCs and MSCs were allocated more independently and the minority of both was gathered. We speculate that TCs might contribute to the migration of MSCs to the lung tissue, survival of MSCs in lung tissue, and therapeutic effects of MSCs on asthma, probably through the alterations of inflammatory cells and mediators. The present study provides a clear evidence that there were synergistic effects of TCs–MSCs co‐transplantation in experimental asthma, although the exact mechanisms are to be furthermore explored.

In conclusion, the present study at first demonstrated that the transplantation of TCs could improve allergen‐induced airway inflammation and hyper‐responsiveness preclinically, with the down‐regulation of Th2‐related cytokine IL‐4, transcription factor GATA‐3 and Th2 cell differentiation, while up‐regulation of Th1‐related cytokine IFN‐γ, transcription factor T‐bet and Th1 cells proliferation. Co‐transplantation of TCs with MSCs showed better therapeutic effects on experimental asthma, even though the therapeutic effects of TCs alone were similar to those of MSCs alone. Thus, our data indicate that the combination of TCs and MSCs can be a new alternative for asthma therapy.

## Conflict of interest

The authors declare that they have no conflict of interest to disclose.

## Supporting information


**Fig. S1** The flow chart of establishment of acute asthma model.

## References

[1] Popescu LM , Faussone‐Pellegrini MS . TELOCYTES – a case of serendipity: the winding way from interstitial cells of Cajal (ICC), *via* interstitial Cajal‐like cells (ICLC) to TELOCYTES. J Cell Mol Med. 2010; 14: 729–40. 20367664 10.1111/j.1582-4934.2010.01059.x PMC3823108

[2] Popescu LM . The tandem: telocytes – stem cells. Int J Biol Biomed Eng. 2011; 5: 83–92.

[3] Faussone‐Pellegrini MS , Gherghiceanu M . Telocyte's contacts. Semin Cell Dev Biol. 2016; 55: 3–8. 26826524 10.1016/j.semcdb.2016.01.036

[4] Popescu LM , Fertig ET , Gherghiceanu M . Reaching out: junctions between cardiac telocytes and cardiac stem cells in culture. J Cell Mol Med. 2016; 20: 370–80. 26538457 10.1111/jcmm.12719 PMC4727556

[5] Boos AM , Weigand A , Brodbeck R , *et al* The potential role of telocytes in tissue engineering and regenerative medicine. Semin Cell Dev Biol. 2016; 55: 70–8. 26805441 10.1016/j.semcdb.2016.01.021

[6] Díaz‐Flores L , Gutiérrez R , Díaz‐Flores L Jr , *et al* Behaviour of telocytes during physiopathological activation. Semin Cell Dev Biol. 2016; 55: 50–61. 26826526 10.1016/j.semcdb.2016.01.035

[7] Galiger C , Kostin S , Golec A , *et al* Phenotypical and ultrastructural features of Oct4‐positive cells in the adult mouse lung. J Cell Mol Med. 2014; 18: 1321–33. 24889158 10.1111/jcmm.12295 PMC4124017

[8] Ibba‐Manneschi L , Rosa I , Manetti M . Telocyte implications in human pathology: an overview. Semin Cell Dev Biol. 2016; 55: 62–9. 26805444 10.1016/j.semcdb.2016.01.022

[9] Bei Y , Zhou Q , Sun Q , *et al* Telocytes in cardiac regeneration and repair. Semin Cell Dev Biol. 2016; 55: 14–21. 26826525 10.1016/j.semcdb.2016.01.037

[10] Shi L , Dong N , Chen C , *et al* Potential roles of telocytes in lung diseases. Semin Cell Dev Biol. 2016; 55: 31–9. 26855021 10.1016/j.semcdb.2016.02.008

[11] Song D , Cretoiu D , Cretoiu SM , *et al* Telocytes and lung disease. Histol Histopathol. 2016; 31: 1303–14. 27463150 10.14670/HH-11-807

[12] Srour N , Thébaud B . Stem cells in animal asthma models: a systematic review. Cytotherapy. 2014; 16: 1629–42. 25442788 10.1016/j.jcyt.2014.08.008

[13] Duffy MM , Ritter T , Ceredig R , *et al* Mesenchymal stem cell effects on T‐cell effector pathways. Stem Cell Res Ther. 2011; 2: 34. 21861858 10.1186/scrt75 PMC3219065

[14] Alkhouri H , Poppinga WJ , Tania NP , *et al* Regulation of pulmonary inflammation by mesenchymal cells. Pulm Pharmacol Ther. 2014; 29: 156–65. 24657485 10.1016/j.pupt.2014.03.001

[15] Zheng Y , Li H , Manole CG , *et al* Telocytes in trachea and lungs. J Cell Mol Med. 2011; 15: 2262–8. 21810171 10.1111/j.1582-4934.2011.01404.x PMC4394233

[16] Yen YT , Yang HR , Lo HC , *et al* Enhancing autophagy with activated protein C and rapamycin protects against sepsis‐induced acute lung injury. Surgery. 2013; 153: 689–98. 23434181 10.1016/j.surg.2012.11.021

[17] Cantarero Carmona I , LuesmaBartolomé MJ , JunqueraEscribano C . Identification of telocytes in the lamina propria of rat duodenum: transmission electron microscopy. J Cell Mol Med. 2011; 15: 26–30. 21054782 10.1111/j.1582-4934.2010.01207.x PMC3822490

[18] Palomares O , Martín‐Fontecha M , Lauener R , *et al* Regulatory T cells and immune regulation of allergic diseases: roles of IL‐10 and TGF‐β. Genes Immun. 2014; 15: 511–20. 25056447 10.1038/gene.2014.45

[19] Nemeth K , Keane‐Myers A , Brown JM , *et al* Bone marrow stromal cells use TGF‐beta to suppress allergic responses in a mouse model of ragweed‐induced asthma. Proc Natl Acad Sci USA. 2010; 107: 5652–7. 20231466 10.1073/pnas.0910720107 PMC2851758

[20] Cho KS , Park MK , Kang SA , *et al* Adipose‐derived stem cells ameliorate allergic airway inflammation by inducing regulatory T cells in a mouse model of asthma. Mediators Inflamm. 2014; 2014: 436476. 25246732 10.1155/2014/436476 PMC4160627

[21] Goodwin M , Sueblinvong V , Eisenhauer P , *et al* Bone marrow‐derived mesenchymal stromal cells inhibit Th2‐mediated allergic airways inflammation in mice. Stem Cells. 2011; 29: 1137–48. 21544902 10.1002/stem.656 PMC4201366

[22] Ratajczak MZ , Ratajczak D , Pedziwiatr D . Extracellular microvesicles (ExMVs) in cell to cell communication: a role of telocytes. Adv Exp Med Biol. 2016; 913: 41–9. 27796879 10.1007/978-981-10-1061-3_3

[23] Popescu LM , Gherghiceanu M , Suciu LC , *et al* Telocytes and putative stem cells in the lungs: electron microscopy, electron tomography and laser scanning microscopy. Cell Tissue Res. 2011; 345: 391–403. 21858462 10.1007/s00441-011-1229-z PMC3168741

[24] Gherghiceanu M , Popescu LM . Cardiomyocyte precursors and telocytes in epicardial stem cell niche: electron microscope images. J Cell Mol Med. 2010; 14: 871–7. 20367663 10.1111/j.1582-4934.2010.01060.x PMC3823118

[25] Drummond‐Barbosa D . Stem cells, their niches and the systemic environment: an aging network. Genetics. 2008; 180: 1787–97. 19087970 10.1534/genetics.108.098244 PMC2600921

[26] Rojas‐Ríos P , González‐Reyes A . Concise review: The plasticity of stem cell niches: a general property behind tissue homeostasis and repair. Stem Cells. 2014; 32: 852–9. 24356972 10.1002/stem.1621

[27] El Maadawi ZM . A tale of two cells: telocyte and stem cell unique relationship. Adv Exp Med Biol. 2016; 913: 359–76. 27796899 10.1007/978-981-10-1061-3_23

[28] Zheng Y , Zhang M , Qian M , *et al* Genetic comparison of mouse lung telocytes with mesenchymal stem cells and fibroblasts. J Cell Mol Med. 2013; 17: 567–77. 23621815 10.1111/jcmm.12052 PMC3822657

[29] Wang J , Ye L , Jin M , *et al* Global analyses of chromosome 17 and 18 genes of lung telocytes compared with mesenchymal stem cells, fibroblasts, alveolar type II cells, airway epithelial cells, and lymphocytes. Biol Direct. 2015; 10: 9. 25888380 10.1186/s13062-015-0042-0 PMC4355521

