# Supplementary figures and images for: Therapeutic roles of telocytes in OVA‐induced acute asthma in mice
Source: J Cell Mol Med. 2017 May 19;21(11):2863–71. doi: 10.1111/jcmm.13199 (PMC5661110; doi:10.1111/jcmm.13199)

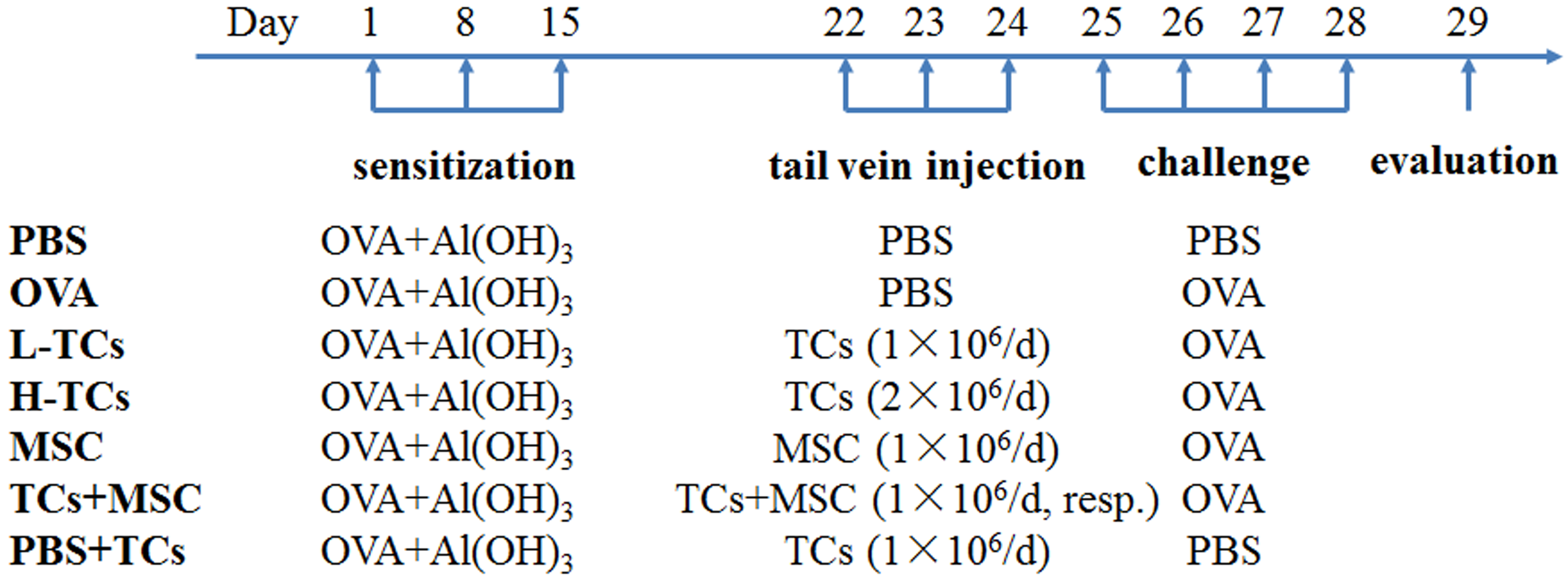

Supplement: Supplementary file 1 — Fig. S1 The flow chart of establishment of acute asthma model. [file JCMM-21-2863-s001.tif]
